# Monocyte/HDL Cholesterol Ratios as a New Inflammatory Marker in Patients with Schizophrenia

**DOI:** 10.3390/jpm13020276

**Published:** 2023-01-31

**Authors:** Nülüfer Kılıç, Gulay Tasci, Seda Yılmaz, Pınar Öner, Sevda Korkmaz

**Affiliations:** 1 Elazığ Fethi Sekin City Hospital, Department of Psychiatry, Elazığ 23100, Türkiye; 2 Elazığ Fethi Sekin City Hospital, Department of Microbiology, Elazığ 23100, Türkiye; 3 Fırat University School of Medicine, Department of Psychiatry, Elazığ 23100, Türkiye

**Keywords:** schizophrenia, monocytes, HDL-C, MHR, inflammation

## Abstract

Purpose: Monocyte/HDL cholesterol ratio (MHR) is a novel inflammatory marker that is used as a prognostic factor for cardiovascular diseases and has been studied in many diseases. The aim of this study was to investigate the role of inflammatory factors in schizophrenia patients by examining MHR levels and to compare schizophrenia patients and healthy controls in terms of cardiovascular disease risk. Method: A total of 135 participants between the ages of 18–65, 85 diagnosed with schizophrenia, and 50 healthy individuals in the control group were included in this cross-sectional study. Venous blood samples were taken from the participants and CBC parameters and lipid profiles were analyzed. The sociodemographic and clinical data form and positive and negative symptoms scale (PANSS) were administered to all participants. Results: Although monocyte levels were significantly higher in the patient group, HDL-C levels were lower at significant levels. MHR was found to be higher in the patient group compared to the control group at significant levels. When compared to the control group, total cholesterol, triglyceride, WBC, neutrophil, basophil, and platelet levels were higher in the patient group at significant levels, and RBC, hemoglobin, and hematocrit levels were significantly lower. Conclusion: The elevated MHR in patients with schizophrenia may contribute to our understanding that inflammation plays important roles in the pathophysiology of schizophrenia. Additionally, knowing the levels of MHR and considering the recommendations, such as diet and exercise, in the treatment approaches made us think that it might be beneficial in protecting schizophrenia patients against cardiovascular diseases and early death.

## 1. Introduction

Schizophrenia is a chronic psychotic disease affecting approximately 1% of the world’s population, beginning in late adolescence or early adulthood generally with positive, negative, and cognitive symptoms [1]. Schizophrenia, a complex disease in which genetic predisposition plays important roles, progresses with neurodegenerative processes and disorders in neurotransmitter systems along with neurodevelopmental deviations. It is already known that exposure to environmental stressors in the early stages of life also plays important roles in the development of schizophrenia. It is considered that exposure to environmental factors from the fetal period may cause neurodevelopmental disorders by increasing the abnormal immune response and increasing the risk of developing schizophrenia [2]. When the broad reflections of the immune response in the organism and its relations with other pathological factors playing roles in schizophrenia are considered, elucidating the role of inflammatory mechanisms in schizophrenia emerges as an important issue. Recent studies suggest that cytokine-mediated inflammatory responses may play an important role in the development of schizophrenia. It is known that the increase in proinflammatory cytokines and the uncontrolled activities of microglia and disorders in neurotransmitter functions affect brain development and cause schizophrenia. [3].

Monocytes and macrophages play important roles in the synthesis and release of pro-inflammatory and prooxidant cytokines [4]. Monocytes, which make up approximately 3–8% of leukocytes in peripheral blood, have important effects in the control of inflammatory processes. It was reported that microglia, which are the monocytes in the brain, can increase inflammatory activation abnormally, affect neuronal growth factors, or be harmful to neurogenesis and synaptogenesis by producing neurotoxic factors and cytokines [5]. Studies conducted on microglia, monocytes, and their products in patient groups and various animal models reported that these cells of the mononuclear phagocyte system play important roles in the pathogenesis of major psychiatric disorders (e.g., schizophrenia, bipolar disorder, and major depressive disorder). It was also reported that activation of microglia and circulating monocytes are elevated in schizophrenia patients and in animal models with schizophrenia-like behaviors, which has important effects on the growth, development, and function of neuronal circuits in the brain [6]. Additionally, it was reported that cytokines, whose blood levels increase after viral or bacterial infections during pregnancy, cross the placenta affecting the brain tissue of the fetus, disrupting the normal neurodevelopmental process, which plays roles in the development of schizophrenia [7].

It was reported in previous studies that high-density lipoprotein cholesterol (HDL-C) protects endothelial cells from the negative effects of low-density lipoprotein cholesterol (LDL-C), inhibiting the oxidation of LDL-C molecules, with antithrombotic, anti-inflammatory, and antioxidant effects [4]. It was also shown that HDL-C reduces the prooxidant and pro-inflammatory effects of monocytes. HDL-C also inhibits the proliferation and differentiation of progenitor cells of monocytes, resulting in decreased monocyte activity. Macrophages differentiating from monocytes take up oxidized LDL-C, and the first foam cells are formed. However, HDL-C molecules remove cholesterol residues from macrophages; in other words, HDL-C molecules exert anti-inflammatory and antioxidant effects through monocytes. For this reason, it is suggested that increased monocytes and decreased HDL-C are associated with inflammation [8]. The relations between macrophage ABCA-1 receptor lipid levels and atherosclerosis were investigated in an experimental study on mice, and it was reported that ABCA-1 deficit increased foam cell accumulation and inflammation; in other words, it had a dual effect on both lipid profile and inflammation suggesting that ABCA1 deficiency is closely related to HDL-C metabolism and inflammation processes [9]. It was reported in a previous study that was conducted in 2020 that there was an increase in the concentrations of atherogenic apoB-containing lipoproteins and a decrease in the concentrations of large HDL-C particles in schizophrenia patients [10]. Additionally, it is already known that schizophrenia patients are prone to dyslipidemia, and antipsychotic drugs may increase the risk of metabolic syndrome, and cardiovascular diseases are increased [11]. It is considered today that the MHR may be a new marker of inflammation and oxidative stress because of the anti-inflammatory and antioxidant effects of HDL-C as well as the pro-inflammatory effect of monocytes [12]. The HDL-C molecule has an anti-inflammatory effect. An increase in monocyte levels indicates increased inflammation. Therefore, an increase in MHR levels indicates increased inflammation. MHR is known both as a marker of inflammation and as a prognostic factor for cardiovascular diseases. MHR has been studied in many medical diseases [13,14,15,16,17,18,19]. However, there are limited studies in the literature on schizophrenia patients [20,21]. For this reason, we preferred to examine the MHR level in this study. In addition, psychiatric diseases and neurological diseases have been detected with artificial intelligence in the literature [1]. Schizophrenia was detected using deep learning techniques. 

### Aims

New studies are important to fully elucidate the role of inflammatory mechanisms, which are increasingly reported in schizophrenia, in understanding the pathophysiology of the disease, to determine the risks of comorbidities, especially cardiovascular disease risks, and to establish effective treatment approaches. Inflammation plays an important role in the etiology of both cardiovascular diseases and schizophrenia. In addition, cardiovascular diseases take the first place among the causes of death in patients with schizophrenia. For this reason, this situation, which causes schizophrenia patients to die in the early period, should be better known, and precautions should be taken in this regard. For these reasons, the aim of this study was to investigate the role of inflammatory factors in schizophrenia patients by examining MHR levels and to compare schizophrenia patients and healthy controls in terms of cardiovascular disease risk.

## 2. Method

### 2.1. Participants and Study Design

A total of 85 people, who were diagnosed with schizophrenia according to the diagnostic criteria of The Diagnostic and Statistical Manual of Mental Disorders-5 (DSM-5), were hospitalized in the Psychiatry Ward of Elazig Fethi Sekin City Hospital, and who met the study criteria, were included in the study randomly. The healthy control group consisted of 50 patients who met the study criteria and were matched with the patient group in terms of age and gender, without any psychiatric, neurological, or metabolic diseases in their past and present history. 

After the purpose and function of the study were explained in detail, written informed consent was obtained from the participants along with a detailed anamnesis. The heights and weights of the participants were measured, and their body mass indices were calculated. Venous blood samples were taken from the participants on an empty stomach, and at the same time of the day, CBC parameters and lipid profile were analyzed. The sociodemographic and clinical data form and positive and negative symptom scale (PANSS) were applied to all patients and the healthy control group. The questionnaire filling process was completed in an average of 30 min. The application and evaluation of the scales were performed by the same psychiatrist. After the scales were filled in, the results of the scales and blood tests were transferred to the electronic medium on a computer on the same day by the relevant psychiatrist and were kept regularly to minimize possible errors and missing data. 

### 2.2. Inclusion and Exclusion Criteria

Inclusion criteria of the patients were being between the ages of 18–65, not having a neurological disease (mental disorders because of brain damage, epilepsy, cerebrovascular disease, dementia, Parkinson’s, etc.) or additional diseases (diabetes, hyperlipidemia, coronary artery disease, malignancy, chronic infection, peripheral artery disease, etc.), and signing the written informed consent form.

For this study, a total of 108 patients were interviewed during the study period, but 4 people were not included in the study due to epilepsy, 8 due to diabetes, 9 due to coronary artery disease, and 3 people because they were illiterate. Although the condition of not having a psychiatric disease was sought in the control group as a criterion, it was considered that the values of individuals with subthreshold psychosis symptoms or individuals with psychotic disease in their close relatives might affect the results. For this reason, the PANSS scale was applied to all participants as it was planned to exclude the patients in the control group with high PANSS values.

The study was conducted in accordance with the principles of the Declaration of Helsinki. Our study was approved by Elazığ Fırat University Clinical Research Ethics Committee (No: 2022/03-30).

### 2.3. Assessment Scales and Inventories

The sociodemographic and clinical data form: This form was prepared by us in line with the clinical experience and information obtained from the sources and considering the aims of the study that was used. This form is a semi-structured form that includes sociodemographic data, such as age, gender, marital status, educational status, occupation, previous prison history, type of crime alleged to have committed, and clinical evaluation questions, such as the presence of psychiatric illness in the family, additional medical illness, and previous psychiatric treatment history.

Positive and negative symptom scale (PANSS): The scale was developed by Kay et al. (1987) as a semi-structured interview scale consisting of 30 items and a 7-point severity assessment. Among these 30 items, 18 were adapted from the brief psychiatry rating scale (BPRS) and 12 from the psychopathology rating scale. Seven items belong to the positive syndrome subscale, 7 to the negative syndrome subscale, and the remaining 16 to the general psychopathology subscale [22]. 

### 2.4. Laboratory Analysis

In the present study, 10 cc venous blood samples were taken from the patients in the patient and control group, after fasting for 8–12 h, into yellow biochemistry tubes and purple blood count tubes. Care was taken to draw blood from all patients at the same time of the day. Then, a complete blood count was analyzed on DXH-800 on the same day, and biochemical parameters were analyzed on Beckman AU-5800 in the biochemistry laboratory of our hospital. Basic hematological parameters, such as monocyte count, were analyzed on an auto analyzer DXH-800 on the same day in the biochemistry laboratory of our hospital, and biochemical parameters (e.g., serum total cholesterol, triglyceride, and HDL-C concentrations) were analyzed on an automatic chemistry analyzer (Beckman AU-5800). Serum low-density lipoprotein cholesterol (LDL-C) values were estimated with the Friedewald Formula or directly measured if triglyceride > 400 mg/dL. The MHR ratio was calculated manually by dividing the monocyte count by HDL-C.

### 2.5. Statistical Analysis

The Statistical Software SPSS for Windows 22 (Statistical Package for Social Sciences for Windows 22) was used to evaluate the data obtained from the participants. Descriptive analyses to give information on the general characteristics of the participants were given as frequency, percentage distribution, and mean ± standard deviation. The data of continuous variables were in the form of mean ± standard deviation. The data on categorical variables were given as n (%). Qualitative variables of the study were given as demographic data, such as gender, age, educational level, socioeconomic status, alcohol, substance use, and whether there was any additional medical disease. Cross-tables and chi-square test were used to evaluate whether there was a relationship between qualitative variables. Quantitative variables were given as the scores obtained from the scales applied to the participants and as blood parameters and MHR values. When evaluating whether there was a relationship between quantitative variables, the independent samples ***t***-test and Pearson correlation analysis were used. When the ***p*** value was calculated at less than 0.05, it was considered statistically significant.

## 3. Results

The present study included 135 participants, 85 of whom were patients diagnosed with schizophrenia according to DSM-5 criteria, and 50 people constituted the healthy control group. The mean age of the patients was 32.77 ± 6.42 (years) and that of the control group was 31.08 ± 7.03 (years), and no significant differences were detected in this respect between the groups (*p* = 0.155). The majority of the participants were male, and it was found that there were no significant gender differences between the patient group and the control group (*p* = 0.613). Although the majority of the patients were literate (*n* = 32, 37.64%), the majority of the control group were university graduates (*n* = 23, 46%) (*p* < 0.001). Most of the patients were not working in an income-generating job (*n* = 45, 52.94%), but all of the control group were working (*n* = 50, 100%) (*p* < 0.001). Additionally, 23.3% of the patients (*n* = 20) had a history of alcohol and substance use, but there was no history of alcohol substance use in the control group (*p* < 0.001). A total of 28.2% (*n* = 24) of the patients had self-mutilation, 17.6% (*n* = 15) of the patients had tattoos, but there were no signs of self-mutilation and tattoos in the control group (*p* < 0.001, *p* = 0.002, respectively). The sociodemographic and clinical data of the participants are given in Table 1.

When the laboratory parameters of the participants were evaluated, monocyte levels were higher in the patient group at a significant level, but HDL-C levels were lower in the patient group at a significant level (*p* = 0.002, *p* = 0.004, respectively). MHR was significantly higher in the patient group when compared to the control group (*p* < 0.001). Total cholesterol, triglyceride, WBC, neutrophil, basophil, and platelet levels were significantly higher in the patient group compared to the control group, (*p* = 0.046, *p* < 0.001, *p* < 0.001, *p* = 0.014, p < 0.001, *p* = 0.004, respectively), and RBC, hemoglobin, and hematocrit levels were significantly lower (*p* = 0.02, *p* = 0.014, *p* = 0.034, respectively) (Table 2). 

When people who had alcohol and substance abuse were excluded from the patient group, monocyte levels were found to be higher in the patient group at significant levels, HDL-C levels were significantly lower in the patient group, and the monocyte/HDL-C ratio was found to be higher in the patient group at a significant level (*p* = 0.009, *p* = 0.007, *p* = 0.001, respectively).

The positive, negative, and general psychopathology subscale scores and total scores of the PANSS scale that were used to measure disease severity were higher in the patient group at a significant level (*p* < 0.001, *p* < 0.001, *p* < 0.001, *p* < 0.001, respectively) (Table 2).

No significant correlations were detected between PANSS scale scores, monocytes, HDL-C, and monocytes/HDL-C ratios. There was a positive correlation between PANSS positive and general psychopathology subscale scores and blood urea levels, a negative correlation between PANSS negative subscale scores and basophil levels, and also a negative correlation between PANSS total scores and RBC levels. (Table 3).

No significant relations were detected between disease duration and MHR (*p* = 0.359).

In logistic regression analysis, monocyte/HDL has an effect on schizophrenia (p < 0.05), and monocyte/HDL increases the disease 1.438 times (Table 4).

**Figure 1:**
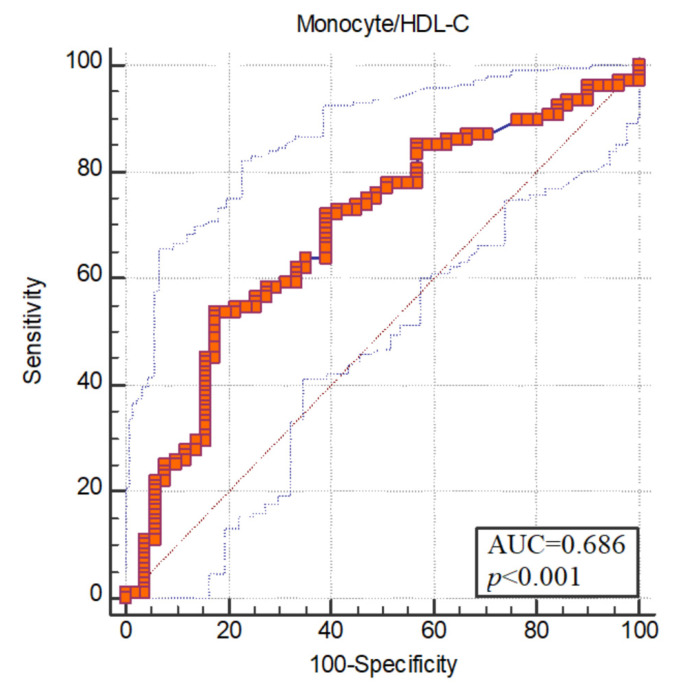
ROC curve.

The areas under the receiver operating characteristic (ROC) curve were found to be statistically significant (*p* < 0.05). ROC curve analysis was used to measure the diagnostic value of the monocyte/HDL-C ratio. The area under the ROC curve of monocyte/HDL for schizophrenia was 0.686. Optimum cut-off value for monocyte/HDL was determined as >0.016. Sensitivity at the breakpoint was 53.70; specificity was set at 82.35 (Figure 1).

## 4. Discussion

In this study, in which cardiovascular disease risk and inflammatory factors were examined according to MHR levels, MHR levels were found to be significantly higher in the schizophrenia patient group. Additionally, monocyte levels were found to be higher in the patient group at significant levels, HDL-C levels were found to be significantly lower in the patient group. When people who had a history of alcohol and substance use were excluded from the patient group, monocyte levels were significantly higher in the patient group, HDL-C levels were significantly lower in the patient group, and MHR levels were significantly higher in the patient group. It was observed that MHR may be associated with an increased risk of schizophrenia.

Studies that investigate the roles of inflammation in schizophrenia reported that the number of monocytes increases, but monocyte functions are impaired and an increased proinflammatory cytokine signal significantly impairs emotional, cognitive, and social functions, increasing sensitivity to environmental risk factors [23]. It was also reported that children living in neighborhoods where violence was intense were at serious risks for psychiatric disorders, which might be mediated by monocyte activation [24]. In a two-year follow-up study examining inflammatory factors, which were considered to be effective on mental and physical development in children living in neighborhoods with high crime rates, it was found that systemic inflammatory response increased in these children, and monocytes mediated this increased response by activating cellular and molecular pathways [25]. In the present study, monocyte levels were significantly higher in the patient group when compared to the control group, which is consistent with the literature data.

Many studies investigate HDL-C ratios in patients with schizophrenia. It was reported in a meta-analysis conducted in schizophrenia patients with first-episode psychosis who did not use medication that HDL-C and total cholesterol ratios were low, and triglyceride ratios were significantly higher [26]. In a one-year follow-up study that was conducted in our country, it was reported that there were increased inflammatory activities in patients with first-episode psychosis, which continued despite the regression in the symptoms of the patients at the end of a one-year follow-up period [27]. It was reported that the increased serum HDL-C levels with the use of antipsychotics that did not cause weight gain for one year after the first episode of psychosis reduced negative symptoms [28]. In a meta-analysis study investigating the effects of pharmacological interventions for the treatment of dyslipidemia because of antipsychotics in patients with schizophrenia, it was reported that the drugs used could decrease LDL-C, triglyceride, and total cholesterol levels while increasing HDL-C levels [29]. In the present study, it was found that HDL-C ratios were significantly lower in patients with schizophrenia, and triglyceride and cholesterol levels were found to be higher, in line with the literature data.

According to recent studies, MHR, which is easy to use, non-invasive, and can be obtained with a simple calculation method, was reported to be a prognostic factor in cardiovascular diseases in addition to being a new inflammatory marker [30]. As the disease progresses in schizophrenia patients, the risk of cardiovascular disease increases because of an unhealthy lifestyle and poor eating habits, as well as the side effects of the antipsychotics used [31]. It is also known that the atypical antipsychotics cause weight gain, hyperlipidemia, diabetes, hypertension, cardiovascular diseases, and premature death. In other words, this risk increases with the course of the disease and the treatments used [32]. It was reported that life expectancy is shorter in schizophrenia patients when compared to the general population, and cardiovascular diseases are among the leading causes of mortality [33]. For this reason, investigating the presence of atherosclerosis in these patients must be determined before the development of coronary heart disease symptoms. In a study investigating atherosclerosis risk factors in schizophrenia patients, it was reported that biochemical parameters, such as P Selectin, IL-6, MCP-1, and CD 40L, which are markers of atherosclerosis, were elevated [34]. In another study investigating arterial stiffness in schizophrenia patients, it was reported that these patients were more prone to develop arterial stiffness because of atherosclerosis, either because of the nature of the disease or the effect of antipsychotic treatment [35]. It was reported in a previous study that examined the relationship between inflammatory factors and cardiovascular diseases that WBC, CRP, and monocyte levels might be risk determinants for cardiovascular diseases [36]. It was shown in another study with cytokines that low-level inflammation increases the cardiometabolic risk [37]. In other words, even if there is no diagnosed coronary heart disease, elevated MHR levels might be an indication that these patients are at serious risk for coronary heart diseases.

MHR was investigated in many diseases in which inflammation is effective. It was reported that it might be a prognostic factor predicting mortality in patients with acute pulmonary embolism [13]. It was also reported that it can be used to predict end-organ damage in hypertensive patients [14]. In another study, it was reported that MHR could be a useful tool for diagnosing obstructive sleep apnoea syndrome (OSAS) and even used for OSAS classification [15]. It was emphasized in another study that MHR rates were lower in women with high physical activity, and MHR could be reduced with physical activity [16]. In another study that evaluated the effects of subclinical hyperthyroidism on lipids and inflammation markers in patients with newly diagnosed polycystic ovary syndrome, MHR was found to be high [17]. In a study that examined MHR in familial Mediterranean fever (FMF) patients, it was reported that a positive correlation was detected between inflammation parameters (e.g., CRP, serum amyloid A, fibrinogen, erythrocyte sedimentation rate, and MHR) [18]. It was also reported that MHR is elevated in patients with acute ischemic stroke and might be an indicator of chronic inflammation [19].

It was found that there are only two studies in the literature examining MHR in schizophrenia patients. In one study, schizophrenic patients with stable coronary disease (Group 1) and without the coronary disease (Group 2) were compared with healthy controls (Group 3), and MHR was found as Group 1 > Group 2 > Group 3. It was reported that MHR is an important independent marker showing inflammation and oxidative stress in patients with schizophrenia and schizophrenia without the stable coronary disease [20]. In another study, 75 schizophrenia patients and 74 healthy controls were compared, and monocyte and MHR values were found to be significantly higher in schizophrenia patients, and a positive correlation was detected between MHR values and age, body mass index, and PANSS scores. However, no significant differences were detected between HDL-C values [21]. In the present study, schizophrenia patients without coronary disease were compared with healthy controls, and MHR values were found to be significantly higher in patients with schizophrenia.

In the present study, when confounding factors, such as alcohol and substance use, were excluded, monocyte and MHR values were significantly higher in patients with schizophrenia, and HDL-C values were significantly lower. There is no study in the literature examining the relations between alcohol, substance use, and MHR. However, it was reported that MHR is elevated in smoking individuals [38].

## 5. Limitations and Strengths

The first was the small sampling size. The second was that it was not evaluated whether the antipsychotics used had effects on MHR. Thirdly, confounding factors, such as smoking, physical activity level, and diet, which might affect the MHR levels, were not evaluated. Additionally, the fact that there were male and female genders and the majority of the participants were males might have had a limited effect on the comparisons in terms of gender. Another limitation was that parameters, such as C-reactive protein and inflammatory cytokines were not studied. These limits the interpretation and generalization of the results. For these findings to gain importance, further studies with larger sample groups are needed.

## 6. Conclusions

The present study is the first one in the literature to investigate the MHR levels in schizophrenic patients without known cardiovascular disease. The elevated MHR, which is a new marker of inflammation in patients with schizophrenia, might contribute to our understanding of the important role of inflammation in the pathophysiology of schizophrenia and cardiovascular disease risk. Additionally, because of the low cost and widespread use of blood count tests, knowing the MHR levels and considering them in the treatment approaches, such as diet and exercise, made us think that it might be beneficial in protecting schizophrenia patients against comorbid diseases, especially cardiovascular disease and early death. In addition, the detection of diseases can be detected by artificial intelligence techniques. In addition, the detection of diseases with OCT, EEG, ECG, MRI and scattergram images can be detected with artificial intelligence techniques [39,40,41,42,43,44,45,46,47,48,49]. In the future, schizophrenia can be detected with inflammatory markers or scattergram images with artificial intelligence techniques.

## Figures and Tables

**Table 1:** Sociodemographic and clinical characteristics of the patient and control groups.

		Patient (*n =* 85)	Control (*n =* 50)	*p* *
		*n* (%)	*n* (%)	
Gender	Female	3 (3.5)	1 (2)	0.613
	Male	82 (96.5)	49 (98)	
Marital status	Single	59 (69.4)	25 (50)	0.005
	Married	19 (22.4)	24 (48)	
	Divorced	7 (8.2)	1 (2)	
Educational status	Literate	32 (37.6)	0 (0)	<0.001
	Primary school	22 (25.9)	0 (0)	
	Secondary school	9 (10.6)	4 (8)	
	High school	10 (11.8)	16 (32)	
	University	3 (3.5)	30 (60)	
	Continues education	9 (10.6)	0 (0)	
Occupation	Military personnel	0 (0)	29 (58)	<0.001
	Healthcare worker	1 (1.2)	17 (34)	
	Worker	32 (37.6)	4 (8)	
	Unemployed	45 (52.9)	0 (0)	
	Self-employment	7 (8.2)	0 (0)	
Prison history	Yes	50 (58.8)	0 (0)	<0.001
	No	35 (41.2)	50 (100)	
Hospitalization	Yes	65 (76.5)	0 (0)	<0.001
	No	20 (23.5)	50 (100)	
History of physical illnesses	Yes	7 (8.2)	0 (0)	0.037
	No	78 (91.8)	50 (100)	
Psychiatric disease in the family	Yes	21 (24.7)	0 (0)	<0.001
	No	64 (75.3)	50 (100)	
Suicide attempt history	Yes	24 (28.2)	0 (0)	<0.001
	No	61 (71.8)	50 (100)	
Disease duration	0	0 (0)	50 (100)	<0.001
	Less than 1 year	33 (38.8)	0 (0)	
	1–10 years	31 (36.5)	0 (0)	
	More than 10 years	21 (24.7)	0 (0)	

Chi-square test was used in the calculations. * *p* < 0.05 was considered statistically significant.

**Table 2:** Scale scores and laboratory parameters of the patient and control groups.

	Patient	Control	*p*
Age (Mean±Standard deviation)	32.77 ± 6.42	31.08 ± 7.04	0.155
Monocyte/HDL-C	0.02 ± 0.01	0.01 ± 0.01	<0.001 *
Glucose (mg/dL)	92.72 ± 23.51	89.56 ± 6.96	0.357
Urea (mg/dL)	26.91 ± 13.56	28.66 ± 6.84	0.399
Creatine (mg/dL)	0.86 ± 0.61	0.86 ± 0.11	0.958
Total Cholesterol (mg/dL)	188.88 ± 48.04	173.02 ± 36.76	0.046 *
HDL-C(mg/dL)	42.29 ± 8.72	46.6 ± 7.39	0.004
LDL-C (mg/dL)	113.41 ± 38.45	107.3 ± 30.16	0.337
TG (mg/dL)	190.08 ± 150.01	108.02 ± 59.62	<0.001 *
WBC (10^3^/uL)	8.7 ± 2.19	7.45 ± 1.60	<0.001 *
RBC (10^6^/uL)	5.07 ± 0.45	5.3 ± 0.35	0.002 *
Hemoglobin (g/dL)	15.30 ± 1.46	15.8 ± 0.73	0.014 *
Hematocrit (%)	44.66 ± 3.88	45.94 ± 2.26	0.034 *
MCV (fL)	87.93 ± 6.81	86.81 ± 3.35	0.278
Platelet(10^3^/uL)	278.35 ± 83.24	239.92 ± 54.41	0.004 *
Neutrophil (10^6^/uL)	5.52 ± 1.80	4.36 ± 1.17	<0.001 *
Lymphocyte(10^3^/uL)	2.26 ± 0.86	2.19 ± 0.63	0.611
Monocyte(10^3^/uL)	0.74 ± 0.27	0.6 ± 0.20	0.002 *
Eosinophil (10^3^/uL)	0.18 ± 0.15	0.21 ± 0.19	0.31
Basophil (10^3^/uL)	0.06 ± 0.05	0.04 ± 0.02	0.016 *
PANSS Positive	26.59 ± 11.86	8.62 ± 1.34	<0.001 *
PANSS Negative	21.58 ± 10.86	9.22 ± 2.630	<0.001 *
PANSS GPP	43.36 ± 19.54	17.08 ± 1.72	<0.001 *
PANSS Total	91.19 ± 34.84	34.92 ± 3.90	<0.001 *
Height (cm)	172.46 ± 7.04	177.84 ± 6.16	<0.001 *
Weight (kg)	76.82 ± 13.17	76.78 ± 11.42	0.985
BMI (kg/m²)	25.85 ± 4.32	24.25 ± 3.219	0.024 *

Abbreviations used in the table: HDL-C: high-density lipoprotein cholesterol, LDL: low-density Lipoprotein cholesterol, WBC: white blood cell (leukocyte) count, RBC: red blood cell (erythrocytes) count, MCV: mean corpuscular volume, BMI: body mass index, PANSS: positive and negative syndrome dcale, GPP: general psychopathology. Independent samples *t* test was used. *p* < 0.05 was considered statistically significant. * Statistically significant.

**Table 3:** Pearson Correlation Analysis of Patients’ Scales and Laboratory Parameters.

		Glucose	Urea	Creatinin	Total Cholesterol	HDL-C	LDL	−CTG	WBC	RBC	Hgb	Hct	MCV	Plt	Neutrophil	Lymphocyte	**Monocyte**	**Eosinophil**	**Basophil**	**MHR**
PANSS Positive	*r*	−0.171	0.265 *	−0.005	−0.134	−0.022	−0.112	−0.068	0.030	−0.210	−0.059	−0.033	0.111	0.042	0.066	−0.103	0.123	0.040	0.020	0.072
	*p*	0.117	0.014	0.964	0.220	0.839	0.309	0.534	0.783	0.054	0.595	0.763	0.313	0.700	0.548	0.350	0.261	0.717	0.854	0.515
PANSS Negative	*r*	0.082	−0.061	−0.024	−0.020	−0.101	0.003	−0.097	0.003	−0.097	−0.108	−0.090	0.032	0.031	0.065	−0.105	0.015	−0.143	−0.251 *	0.043
	*p*	0.454	0.578	0.827	0.858	0.358	0.978	0.378	0.977	0.379	0.327	0.412	0.773	0.776	0.555	0.338	0.889	0.191	0.021	0.698
PANNS GPP	*r*	−0.105	0.224 *	−0.016	−0.089	−0.074	−0.068	−0.086	0.057	−0.212	−0.085	−0.106	0.075	0.130	0.043	0.007	0.106	0.133	−0.095	0.107
	*p*	0.340	0.039	0.883	0.418	0.500	0.537	0.436	0.602	0.051	0.439	0.334	0.492	0.236	0.695	0.948	0.335	0.224	0.385	0.328
PANSS Total	*r*	−0.093	0.206	−0.017	−0.106	−0.078	−0.080	−0.100	0.048	−0.220 *	−0.101	−0.096	0.092	0.101	0.071	−0.059	0.102	0.047	−0.123	0.094
	*p*	0.399	0.059	0.879	0.336	0.478	0.469	0.364	0.662	0.043	0.357	0.382	0.404	0.356	0.518	0.589	0.353	0.668	0.261	0.393

Abbreviations used in the table: HDL-C: high-density lipoprotein cholesterol, LDL-C: low-density lipoprotein cholesterol, TG: triglyceride, WBC: white blood cell (leukocyte) count, RBC: red blood cell (erythrocytes) count, Hgb: hemoglobin, Hct: hematocrit, MCV: mean corpuscular volume, Plt: platelet, MHR: monocyte/HDL-C ratio. Pearson correlation coefficient was used in the calculations. Values shown in the table; ‘*r*’ and ‘*p*’ values. * *p* < 0.05 was considered statistically significant.

**Table 4:** Logistic Regression Analysis of the Effect of Monocyte/HDL-C on Schizophrenia.

	B	S.E.	Sig.	Exp(B)	95% C.I.for EXP(B)	
					Lower	Upper
Monocyte/HDL-C	94.769	31.663	0.003	1.438	1.667	1.287
Constant	−0.701	0.491	0.154	0.496		
Cox & Snell R^2^ = 0.069; Nagelkerke R^2^ = 0.095						

## Data Availability

The data are not publicly available.
